# Randomized, double-masked, sham-controlled trial of efficacy and safety of quantum molecular resonance for treating meibomian gland dysfunction

**DOI:** 10.1038/s41433-025-03890-3

**Published:** 2025-06-27

**Authors:** Lita Uthaithammarat, Ngamjit Kasetsuwan, Usanee Reinprayoon, Yuda Chongpison, Wiyada Quanchareonsap, Paphaphat Dissaneevate

**Affiliations:** 1 Department of Ophthalmology, Faculty of Medicine, Chulalongkorn University and King Chulalongkorn Memorial Hospital, Bangkok, Thailand; 2 Center of Excellence for Cornea and Stem Cell Transplantation, Department of Ophthalmology, Faculty of Medicine, Chulalongkorn University, Bangkok, Thailand; 3 Center of Excellence in Biostatistics, Research Affairs, Faculty of Medicine, Chulalongkorn University, Bangkok, Thailand; 4 Faculty of Medicine, Chulalongkorn University, Bangkok, Thailand

**Keywords:** Eye manifestations, Corneal diseases

## Abstract

**Background:**

This randomized, double-blind, sham-controlled trial aimed to evaluate the novel quantum molecular resonance (QMR) device for meibomian gland dysfunction (MGD) treatment.

**Methods:**

Eighty participants diagnosed with MGD were randomized into QMR or sham-QMR groups. Each procedure was performed on days 0, 7, 14, and 21. Primary (meibum quality score) and other secondary outcomes were examined at baseline and weeks 7 and 11. Tear osmolarity and interleukin (IL)-6 and IL-1 receptor agonist levels were evaluated at baseline and week 7. Adverse events were recorded. A multilevel mixed-effect linear regression model was used for data analysis.

**Results:**

Meibum quality (*p* = 0.008), corneal/conjunctival fluorescein staining score (*p* = 0.036), telangiectatic vessel area (*p* = 0.008), and superior (*p* = 0.011) and inferior (*p* = 0.020) lid meiboscale were significantly improved in the QMR group than those in the sham-treated group at week 11. Superior lid meiboscale (*p* = 0.027) and meibomian gland plugging grade (MGPG) (*p* = 0.017) were significantly improved in the QMR group at week 7. In the QMR group, Ocular Surface Disease Index (OSDI) scores and lid margin thickening grades were significantly lesser at weeks 7 (*p* = 0.002 and <0.001, respectively) and 11 (both *p* < 0.001) than the baseline values. At week 7, IL-6 levels were significantly decreased only in the QMR group (*p* = 0.016). All other parameters did not significantly differ. No serious adverse event occurred.

**Conclusions:**

The QMR device was effective for MGD treatment, with improvements in meibum quality, corneal/conjunctival staining, telangiectatic vessels, MGPG, superior and inferior lid meiboscale, and decreased OSDI score, lid margin thickening grade, and tear IL-6 level.

## Introduction

Meibum is an essential component of the tear film, protecting it from hyperevaporation. Meibomian gland dysfunction (MGD), a chronic abnormality of lipid secretion from meibomian glands in the upper and lower eyelids, is characterized by duct obstruction or qualitative/quantitative changes in meibum secretion. Abnormal meibum secretion may lead to evaporative dry eye disease, which is the most common type of dry eye [1]. Patients’ symptoms vary from no symptoms to ocular discomfort, redness, itching, or photophobia. If these conditions are left untreated, inflammation can occur and damage the ocular surface.

Currently, a gold-standard MGD treatment has not been established; however, recommendations include warm compressions and lid massages to liquefy the meibum, reopen the obstructed gland, and remove the meibum. Moreover, anti-inflammatory agents are used to decrease inflammation and improve meibum quality [2]. In addition, various novel device-based treatments are available. A quantum molecular resonance (QMR)-based device was recently suggested as an effective treatment for MGD and dry eye disease [3–7]. QMR is a physical effect that occurs when tissues are stimulated with weak alternating electric current patterns at 4–64 MHz. This randomized sham-controlled trial aimed to ascertain the efficacy, safety, and mechanisms of MGD treatment using this QMR device.

## Methods

This study was approved by the Institutional Review Board of the Faculty of Medicine, Chulalongkorn University, Thailand, and adhered to the tenets of the Declaration of Helsinki. This trial was registered at ClinicalTrials.gov (NCT05165342). Written informed consent was obtained from all participants. Additionally, written informed consent for the publication of identifiable patient photographs (Supplementary Fig. 1B) was obtained from the patient prior to publication.

Participants diagnosed with MGD according to the “International Workshop on Meibomian Gland Dysfunction: Report of Subcommittee on Management and Treatment of Meibomian Gland Dysfunction” [2] at the outpatient clinic, Department of Ophthalmology, King Chulalongkorn Memorial Hospital, Thailand, were recruited between December 10, 2021, and April 30, 2022. The inclusion criteria were (1) age 18–80 years, (2) ability and willingness to comply with the treatment/follow-up schedule, and (3) stage 1–4 MGD diagnosis in one or both eyes. Exclusion criteria are presented in Supplementary Data.

The sample size was calculated using the meibum quality score as the primary outcome with 80% power (β = 0.2). Eleven participants were required for each of the QMR and sham-QMR groups to detect a clinically significant difference in five scores between these groups. With a two-sided statistical significance level of 5% (α = 0.05), the mean meibum quality score and pooled standard deviation (SD) were calculated as 13.8 and 4.10 [4, 8].

### Experimental design

This was a prospective, randomized, double-blind, sham-controlled clinical trial. In the randomization step, a treatment sequence was randomly permuted in blocks of six and eight, with block sizes allocated unequally in a ratio of 1:4:6:4:1 (Pascal’s triangle) for the first 34 participants. Subsequently, minimization [9] was used to assign participants to the sham-QMR or QMR group according to sex, age (≤60 or >60 years), and MGD stage (1/2 or 3/4). The unit of randomization/minimization was the individual participant. The sequences were placed in an opaque, sealed envelope and stored by a researcher (W.Q.). All investigators and participants were blinded to the treatment assignments for the duration of their involvement. Upon study completion, a research assistant unmasked the randomization sequence and forwarded the results to the study team for data analysis. A single evaluator (L.U.) examined patients throughout the study.

### Treatment

This study used the QMR Rexon-Eye device (Resono Ophthalmic, Trieste, Italy). All participants underwent either QMR or sham-QMR treatment performed by a single research assistant on days 0, 7, 14, and 21. The QMR treatment protocol was recommended by the company that developed the Rexon-Eye device. The treatment was administered using goggles for 20 min per visit and applied to the closed upper and lower eyelids. The intensity was set at 5 (custom units), corresponding to an average power of 12 W, with 60 V voltage and 200 mA current between the goggle electrode and the neutral electrode attached to the participant’s chair (Supplementary Fig. 1). The sham-QMR group received the same treatment except that the power was set to zero. During the procedure, sound was perceived the same in the QMR and sham-QMR groups. All participants were required to use only preservative-free artificial tears (0.18% sodium hyaluronate) at least four times daily, together with lid hygiene (lid scrub and warm compression) twice daily. Their compliance was checked by a research assistant.

### Clinical assessment

For each participant, the eye with the higher MGD stage was evaluated. Primary (meibum quality score) and secondary (Ocular Surface Disease Index [OSDI] score, tear meniscus height [TMH, mm], noninvasive tear break-up time [NITBUT, s], bulbar conjunctival hyperemia [grade], tear film lipid layer thickness [TFLLT, nm], corneal and conjunctival fluorescein staining score, Schirmer’s test [mm], area of lid telangiectasia [pixel], lid margin thickening and irregularity grade, meibomian gland plugging grade [MGPG], superior and inferior lid meiboscale, and meibum expressibility grade) outcomes were examined at baseline, 7-week (1 month after the last treatment), and 11-week (2 months after the last treatment) visits. Tear osmolarity (mOsm/L) and interleukin (IL)-1 receptor agonist (IL-1Ra) and IL-6 levels (pg/mL) were evaluated at baseline and 7-week visits. Best-corrected visual acuity (BCVA), uncorrected visual acuity (UCVA), and intraocular pressure (IOP, mmHg) were recorded on days 0, 7, 14, and 21, week 7, and week 11. The sequence and details of the clinical assessment are provided in Supplementary Data.

### Statistical analysis

Demographics and baseline clinical characteristics are reported as mean with SD, median with first and third quartiles, or frequency with percentage. Linear mixed model regression with an adjusted baseline was used to analyze all parameters over time. The results are presented as estimated means and 95% confidence intervals (CIs). To determine whether each outcome measure in the two groups changed differently over time, a time–treatment group interaction term was included if *p*_interaction_ was <0.2. To control for type 1 errors, Scheffé’s method was used as a post hoc test for multiple comparisons. The baseline cytokine concentration was described as a geometric mean with a percent coefficient of variation (%CV). Normal distribution was assessed with a histogram and the Shapiro–Wilk test after logarithmic transformation. Linear mixed model regression with adjusted log-transformed baseline cytokine value was used to evaluate differences in logarithmic mean values of cytokine concentrations. Intention-to-treat analyses of efficacy outcomes were conducted. For all analyses, statistical significance was set at an alpha level of 0.05. Stata version 15.1 was used (StataCorp, College Station, TX).

## Results

**Fig. 1:**
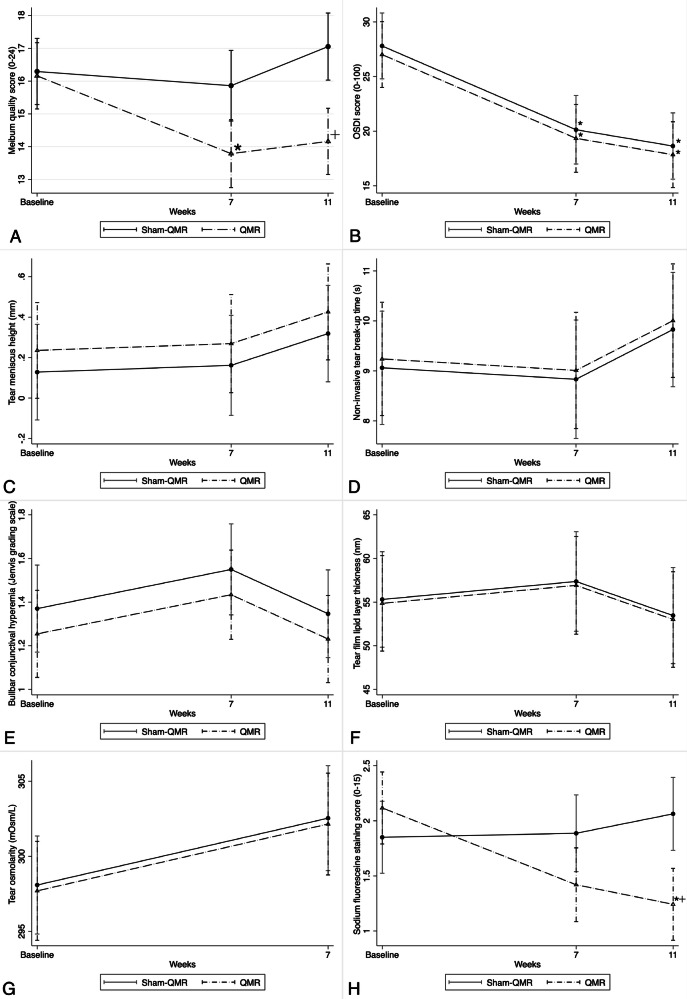
Eight outcomes in the QRM and sham-QRM groups at baseline and 7- and 11-week follow-ups. **A** Meibum quality score, **B** Ocular Surface Disease Index score, **C** tear meniscus height, **D** noninvasive tear break-up time, **E** bulbar conjunctival hyperemia, **F** tear film lipid layer thickness, **G** tear osmolarity, and **H** sodium fluorescein staining score. + *p* < 0.05, comparison between the two study groups; * *p* < 0.05, comparison with baseline within each study group. QMR quantum molecular resonance.

**Fig. 2:**
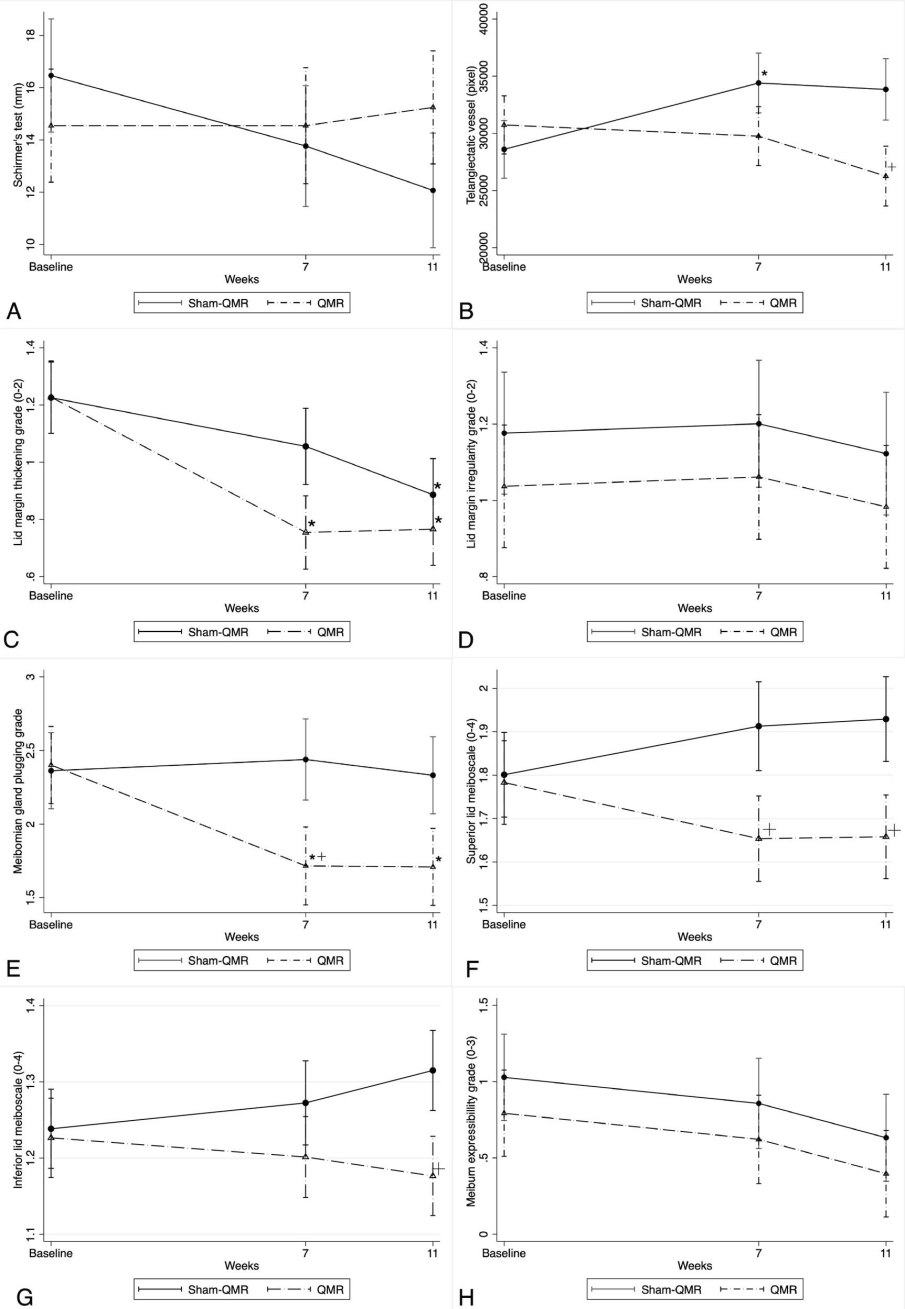
Eight outcomes in the QRM and sham-QRM groups at baseline and 7- and 11-week follow-ups. **A** Schirmer’s test, **B** area of telangiectasia vessels, **C** lid margin thickening grade, **D** lid margin irregularity grade, **E** meibomian gland plugging grade, **F** superior lid meiboscale, **G** inferior lid meiboscale, and **H** meibum expressibility grade. + *p* < 0.05, comparison between the two study groups; * *p* < 0.05, comparison with baseline within each study group. QMR quantum molecular resonance.

Of 80 eligible participants, seven were lost to follow-up (Supplementary Fig. 2). The baseline characteristics and clinical and laboratory parameters of the QMR and sham-QMR groups are shown in Supplementary Table 1. All estimated outcomes calculated from linear mixed models are shown in Table 1, Figs. 1 and 2.

**Table 1:** All clinical estimated mean outcomes at baseline, 1 month after the last treatment (week 7), and 2 months after the last treatment (week 11).

Outcome (95% confidence interval)	Group	Baseline	1 month after the last treatment	1 month vs baseline	QMR vs Sham (1 month)	2 months after the last treatment	2 months vs baseline	QMR vs Sham (2 months)
OSDI score (0–100)	QMR	27.01; [24.00, 30.02]	19.34; [16.24, 22.45]	*P* = 0.002*	*P* = 0.161	17.84; [14.83, 20.86]	*P* < 0.001*	*P* = 0.999
	Sham-QMR	27.80; [24.79, 30.82]	20.13; [16.99, 23.27]	*P* = 0.002*		18.63; [15.61, 21.67]	*P* < 0.001*	
TMH (mm)	QMR	0.24; [−0.001, 0.47]	0.27; [0.03, 0.51]	*P* = 1.000	*P* = 0.979	0.43; [0.19, 0.66]	*P* = 0.893	*P* = 0.979
	Sham-QMR	0.12; [−0.11, 0.37]	0.16; [−0.09, 0.41]	*P* = 1.000		0.32; [0.08, 0.56]	*P* = 0.893	
NITBUT (s)	QMR	9.24; [8.11, 10.37]	9.01; [7.85, 10.17]	*P* = 1.000	*P* = 1.000	10.00; [8.87, 11.14]	*P* = 0.944	*P* = 1.000
	Sham-QMR	9.06; [7.93, 10.20]	8.83; [7.65, 10.02]	*P* = 1.000		9.83; [8.68, 10.97]	*P* = 0.944	
Bulbar conjunctival hyperemia (JENVIS scale, 0–4)	QMR	1.25; [1.06, 1.45]	1.43; [1.23, 1.64]	*P* = 0.847	*P* = 0.940	1.23; [1.03, 1.43]	*P* = 1.000	*P* = 0.940
	Sham-QMR	1.37; [1.17, 1.57]	1.55; [1.34, 1.76]	*P* = 0.847		1.35; [1.15, 1.55]	*P* = 1.000	
Tear film lipid layer thickness (nm)	QMR	54.86; [49.40, 60.32]	56.92; [51.33, 62.51]	*P* = 0.996	*P* = 1.000	53.01; [47.54, 58.48]	*P* = 0.997	*P* = 1.000
	Sham-QMR	55.31; [49.84, 60.77]	57.36; [51.67, 63.07]	*P* = 0.996		53.45; [47.95, 58.96]	*P* = 0.997	
Tear osmolarity (mOsm/L)	QMR	297.70; [294.40, 300.99]	302.15; [298.76, 305.54]	*P* = 0.169	*P* = 0.998	-	-	-
	Sham-QMR	298.09; [294.82, 301.35]	302.54; [299.05, 306.03]	*P* = 0.169		-	-	
Sodium fluorescein staining score (0–15)	QMR	2.12; [1.79, 2.44]	1.42; [1.09, 1.75]	*P* = 0.114	*P* = 0.610	1.24; [0.92, 1.57]	*P* = 0.013*	*P* = 0.036*
	Sham-QMR	1.85; [1.52, 2.18]	1.89; [1.54, 2.24]	*P* = 1.000		2.06; [1.73, 2.39]	*P* = 0.975	
Schirmer’s test (mm)	QMR	14.55; [12.39, 16.71]	14.54; [12.33, 16.77]	*P* = 1.000	*P* = 0.999	15.25; [13.09, 17.41]	*P* = 0.999	*P* = 0.545
	Sham-QMR	16.47; [14.30, 18.63]	13.77; [11.46, 16.08]	*P* = 0.686		12.07; [9.88, 14.26]	*P* = 0.121	
Telangiectatic vessel (pixel)	QMR	30,732.69; [28,188.34, 33,277.04]	29,755.25; [27,177.81, 32,332.70]	*P* = 0.997	*P* = 0.297	26,259.85; [23,646.30, 28,873.40]	*P* = 0.231	*P* = 0.008*
	Sham-QMR	28,600.29; [26,082.36, 31,118.22]	34,406.38; [31,793.07, 37,019.69]	*P* = 0.039*		33,839.81; [31,155.88, 36,523.75]	*P* = 0.102	
Lid margin thickening grade (0–2)	QMR	1.23; [1.10, 1.35]	0.75; [0.63, 0.88]	*P* < 0.001*	*P* = 0.070	0.76; [0.64, 0.89]	*P* < 0.001*	*P* = 0.885
	Sham-QMR	1.23; [1.10, 1.35]	1.06; [0.92, 1.19]	*P* = 0.602		0.89; [0.76, 1.01]	*P* = 0.009*	
Lid margin irregularity grade (0–2)	QMR	1.04; [0.88, 1.20]	1.06; [0.90, 1.22]	*P* = 1.000	*P* = 0.769	0.98; [0.82, 1.14]	*P* = 0.997	*P* = 0.769
	Sham-QMR	1.18; [1.02, 1.34]	1.20; [1.03, 1.37]	*P* = 1.000		1.12; [0.96, 1.28]	*P* = 0.997	
MG plugging grade (0–3)	QMR	2.40; [2.14, 2.66]	1.72; [1.45, 1.98]	*P* = 0.009	*P* = 0.017*	1.71; [1.45, 1.97]	*P* = 0.007	*P* = 0.054
	Sham-QMR	2.36; [2.10, 2.62]	2.44; [2.16, 2.71]	*P* = 0.999		2.33; [2.07, 2.59]	*P* = 1.000	
Superior lid meiboscale (0–4)	QMR	1.78; [1.69, 1.88]	1.65; [1.55, 1.75]	*P* = 0.454	*P* = 0.027*	1.66; [1.56, 1.75]	*P* = 0.475	*P* = 0.011*
	Sham-QMR	1.80; [1.70, 1.90]	1.91; [1.81, 2.02]	*P* = 0.653		1.93; [1.83, 2.03]	*P* = 0.460	
Inferior lid meiboscale (0–4)	QMR	1.23; [1.17, 1.28]	1.20; [1.15, 1.25]	*P* = 0.987	*P* = 0.658	1.18; [1.12, 1.23]	*P* = 0.772	*P* = 0.020*
	Sham-QMR	1.24; [1.19, 1.29]	1.27; [1.22, 1.33]	*P* = 0.957		1.31; [1.26, 1.37]	*P* = 0.323	
Meibum quality score (0–24)	QMR	16.16; [15.15, 17.17]	13.79; [12.75, 14.82]	*P* = 0.034*	*P* = 0.193	14.16; [13.15, 15.17]	*P* = 0.116	*P* = 0.008*
	Sham-QMR	16.30; [15.28, 17.30]	15.86; [14.78, 16.94]	*P* = 0.996		17.06; [16.03, 18.08]	*P* = 0.939	
Meibum expressibility grade (0–3)	QMR	0.79; [0.51, 1.08]	0.62; [0.33, 0.91]	*P* = 0.970	*P* = 0.761	0.40; [0.11, 0.68]	P = 0.411	*P* = 0.761
	Sham-QMR	1.03; [0.75, 1.31]	0.85; [0.56, 1.15]	*P* = 0.970		0.63; [0.35, 0.92]	*P* = 0.411	
Tear Cytokine (log-pg/ml)								
IL-6 level	QMR	3.23; [2.95, 3.52]	2.73; [2.44, 3.02]	*P* = 0.016	*P* = 0.146	-	-	-
	Sham-QMR	3.10; [2.82, 3.38]	3.05; [2.74, 3.35]	*P* = 0.806		-	-	
IL-1Ra level	QMR	12.45; [12.20, 12.70]	12.33; [12.06, 12.59]	*P* = 0.498	*P* = 0.106	-	-	-
	Sham-QMR	12.39; [12.14, 12.64]	12.64; [12.06, 12.59]	*P* = 0.194		-	-	

*IL* interleukin, *NITBUT* noninvasive tear break-up time, OSDI Ocular Surface Disease Index, *TMH* tear meniscus height

* *P* < 0.05

### Primary outcome: meibum quality

The meibum quality score was significantly lower in the QMR group than that in the sham-QMR group at 11 weeks (*p* = 0.008). In the QMR group, the 7-week score was significantly lower than that at baseline (*p* = 0.034). All other pairwise comparisons showed no significant group differences.

### Secondary outcomes

**Fig. 3:**
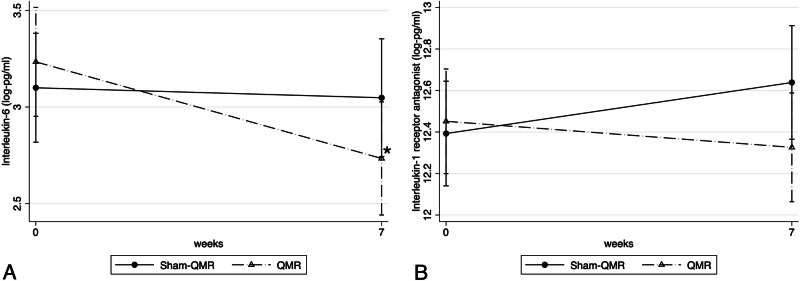
Logarithmic mean values of tear cytokine levels in the QRM and sham-QRM groups at baseline and 7-week follow-up. Levels of (**A**) interleukin-6 and (**B**) interleukin-1 receptor antagonist in tear fluid. * *p* < 0.05, comparison with baseline within each study group. QMR quantum molecular resonance.

All secondary outcomes were presented in Table 1, Figs. 1 and 2. The area of lid telangiectasia and tear cytokine levels are presented in Supplementary Fig. 3 and Fig. 3, respectively.

### Safety profile

#### BCVA, UCVA, and IOP

Pairwise comparisons of these parameters at each time point were not significantly different (Supplementary Table 2), and the ocular examinations of all participants were normal throughout.

#### Adverse events

After the first QMR treatment, three participants of the QMR group experienced mild upper eyelid redness, which resolved without treatment within 3 days. The reason was that the goggles were too tight, and no further complaints were reported.

#### Temperature of the upper eyelid skin

The mean temperatures at baseline and on days 7, 14, and 21 are presented in Supplementary Table 3. The estimated mean difference between the temperatures before and after treatment was significantly higher in the QMR than in the sham-QMR group on days 0, 7, 14, and 21 (all *p* < 0.001; Table 2).

**Table 2:** Estimated mean difference with 95% confidence interval of the upper eyelid temperature between immediately after and just before the intervention.

Difference of Temperature (After-Before, Celsius)	QMR	Sham-QMR	*P*
Day 0	1.67; [1.39, 1.95]	0.27; [-0.04, 0.51]	<0.001
Day 7	1.46; [1.19, 1.74]	0.03; [-0.24, 0.31]	<0.001
Day 14	1.77; [1.50, 2.05]	0.34; [0.06, 0.61]	<0.001
Day 21	1.50; [1.22, 1.77]	0.06; [-0.21, 0.34]	<0.001

## Discussion

This is the first prospective, randomized, double-blind, sham-controlled trial comparing QMR with sham-QMR treatment for MGD. We found that QMR treatment significantly improved MGD-related signs compared to the sham-QMR group, including meibum quality, corneal and conjunctival staining, lid margin telangiectasia, MGPG, and lid meiboscale. In the QMR group, OSDI scores, lid margin thickening grades, and tear IL-6 levels were significantly improved after treatment compared to the baseline values.

Electrical stimulation with low-intensity currents has demonstrated successful outcomes in tissue regeneration [10–13]. The meibomian glands’ regenerative potential has been previously reported using topical diquafosol [14], intraductal meibomian gland probing [15], and lid hygiene [16]. This suggests that QMR can induce their regeneration. The QMR’s specific current pattern differs from that of other transcutaneous electrical stimulation (TES) approaches [17, 18]. This study showed that meibomian gland function and structure significantly improved after QMR treatment. Improvement in corneal staining indicates a decrease in ocular surface inflammation, showing reversibility of corneal epithelium damage [19]. Improvements in meiboscale, meibum quality, and meibomian gland plugging, which is positively correlated with meibomian gland dropout [20], suggested the reversibility of meibomian gland damage. These findings are consistent with the theory that electrical stimulation facilitates tissue regeneration.

Additionally, QMR alleviates inflammation as demonstrated by decreased matrix metalloproteinase (MMP)-9 levels [3] and leukocyte infiltration in tissues [10] reported by other studies and reductions in IL-6 levels with a trend toward decreasing IL1-Ra levels according to our study.

The meibomian gland is not only regulated by hormones but is also influenced by nerve stimulation, with nerve density playing a crucial role in its function [21]. The alternating current patterns of QMR may stimulate nerves innervating meibomian glands, potentially leading to nerve and subsequent glandular regeneration.

A study examining QMR effects on human mesenchymal stromal cells revealed its impact on transcriptional regulation by modulating genes associated with developmental processes and cellular pathways, including metabolism, kinase activity, and cellular regulation. Real-time polymerase chain reaction showed significantly increased *MMP1*, *PLAT*, and *ARHGAP22* expression after QMR treatment, whereas *A2M* expression was significantly decreased [22]. These genes are involved in extracellular matrix remodeling, angiogenesis, embryogenesis, inflammation reduction, and wound healing promotion. The impact at transcriptional and cellular levels may decrease inflammatory cytokine levels, e.g., IL1-Ra and IL-6 levels, and improve ocular surface staining, as indicated by our results. A previous study showed a strong association between ocular surface staining and ocular surface inflammation [23]. The QMR effects on transcriptional regulation suggest that its effectiveness may persist for an extended period after treatment.

The meibum quality score was significantly lower in the QMR group at 11 weeks than that in the sham-QMR group and at 7 weeks than the baseline values. At week 7, MGPG was much lower in the QMR group than MGPG in the sham-QMR group. Moreover, MGPG in the QMR group was significantly improved at week 7 than the baseline values. This aligns with the findings of previous studies [3, 4]. Additionally, the superior and inferior lid meiboscales were significantly improved in the QMR group. Likewise, Kavroulaki et al. [6] reported such improvements 2 months after the last treatment; this effect persisted for at least 2 months after treatment in our study. In contrast, Trivli et al. found no change in meiboscale results [3].

Interestingly, meibum expressibility reflecting meibum secretion [24] did not improve in our study. Meibum lipids contribute to tear film stability and protect tears from hyperevaporation [24]. Accordingly, NITBUT, reflecting tear film stability, and TFLLT, reflecting the amount of lipids in the tear film, did not improve after treatment in our study. This aligns with the findings of Foo et al. [7]; however, other studies reported different findings [3, 4, 6, 17]. Although meibum quality was significantly improved, meibum expressibility was unchanged after treatment in our study. Meibum quality was reported to be a sensitive and specific test for MGD, whereas meibum expressibility has poor efficacy owing to variable secretory activity of individual glands depending on their location [25]. NITBUT and TFLLT may require longer follow-up periods to detect changes.

In both study groups, patient-reported dry eye symptoms reflected by OSDI scores significantly improved at 7- and 11-week follow-ups compared with the baseline values without significant group differences. The application of artificial tears with lid hygiene (standard treatment) may have caused this. The OSDI and Standard Patient Evaluation of Eye Dryness Questionnaire scores were also significantly improved in previous QMR [3–7] and TES [17] studies. Moreover, the OSDI score primarily reflects the patients’ subjective perceptions. Notably, the patients were treatment-naïve, and they compared their symptoms before and after receiving the standard treatment together with the intervention (either QMR or sham-QMR), resulting in improved scores. Several studies have reported an inconsistent association between clinical signs and subjective symptoms of dry eye disease [26–29], and in this study, the sham-QMR group similarly exhibited symptomatic improvement despite the absence of significant changes in clinical signs.

According to our findings, QMR alleviated inflammation in patients with MGD. IL-6 levels were significantly decreased at week 7, with a decreasing trend in IL1-Ra levels after treatment. Trivli et al. reported that QMR treatment significantly reduced tear MMP-9 levels [3]. Moreover, IL-6, IL1-Ra, and MMP-9 are associated with evaporative dry eye, including MGD [30–32]. Abnormalities in meibomian gland secretion contribute to tearing hyperosmolarity and further induce stress to the ocular surface, in which epithelial cells express IL-6. Furthermore, IL-6 amplifies the process by recruiting both innate and adaptive immune cells to the site of inflammation, promoting the vicious cycle of dry eye disease and ocular surface inflammation [33]. Additionally, ocular surface inflammation contributes to MGD [34]. In a previous study, IL-6 levels were significantly associated with MGD and non-Sjögren aqueous tear deficiency [30, 35]. As QMR can decrease IL-6 levels, it may eliminate one of the factors leading to the vicious cycle of dry eye and MGD. IL1-Ra is a natural antagonist of the pro-inflammatory cytokine IL1β by competitively binding to type 1 IL1 receptors. The spare receptor effect causes IL1-Ra levels to be higher than IL1β levels in normal tear fluid [36]. Moreover, IL1-Ra tear levels were higher in patients with MGD than in healthy participants [36], and they were nonsignificantly decreased after applying QMR, which led us to infer that QMR may mitigate IL1-Ra levels. Foo et al. found nonsignificant changes in the levels of 11 cytokines, including IL-6, in tears of patients with dry eye [7]. Desiccating dry eye stress increased the number of sloughing and stained corneal cells [37]. Although in our study IL-6 and IL1-Ra levels decreased in week 7, corneal and conjunctival fluorescein staining significantly improved in 11 weeks. Inflammation-associated clinical signs improve 1 month after cytokine reduction. Five previous TES/QMR studies showed significant improvements in corneal and conjunctival fluorescein levels in the treatment group [3–5, 7, 17]. However, bulbar conjunctival hyperemia was not significantly changed in our study. Lid telangiectasia is an MGD characteristic and reflects exposure to insults, including inflammation [38, 39]. Our findings showed the efficacy of QMR treatment in decreasing lid margin telangiectasia areas at 7 and 11 weeks, whereas in the sham-QMR group, this area was significantly increased at 7 weeks. The QMR-induced amelioration of inflammation supports the hypothesis that QMR can improve lid margin telangiectasia.

It was hypothesized that QMR promotes tear secretion [40]. Previous studies demonstrated QMR effectiveness in increasing Schirmer’s test results [4, 5] and improving TMH [6]. However, others found that QMR did not affect TMH or Schirmer’s test results [3, 7, 17]. According to Han et al., tear osmolarity significantly improved at 12 weeks [17]. Our study found no significant improvements in TMH, Schirmer’s test results, or tear osmolarity in the QMR group, whereas Schirmer’s test was worse in the sham-QMR group at 11 weeks. We postulate that longer follow-up or more QMR treatments are needed to detect significant improvements in Schirmer’s test and other tear volume-associated outcomes because Schirmer’s test results were improved after a 12-month follow-up [5].

Lid margin thickening is reportedly associated with MGD-related meibomian gland morphological features except for meibomian gland dropout [20]. In our study, lid margin thickening was significantly improved at 7 and 11 weeks in the QMR group and at 11-week follow-up in the sham-QMR group. The improvement in the sham-QMR group might be due to lid hygiene, but our results suggest that QMR treatment with lid hygiene decreases lid thickening more effectively and faster than lid hygiene alone. Conflicting results have been reported regarding the association between MGD and lid margin irregularity [20, 41, 42]. In our study, lid margin irregularity did not change after QMR treatment.

By contrast, thermal pulsation proximal to a distal peristaltic motion device introduces heat of 42.5 °C to the eyelid. Thermal pulsation for MGD treatment involves liquefying and evacuating meibum. The positive effect persisted for 3 months in one study and for 12 months in another [43]. However, patients with a short fornix and small palpebral aperture size reported discomfort and soreness after treatment [43]. Another novel approach to effectively treating MGD is IPL therapy. Underlying mechanisms include reducing inflammation, thrombosing telangiectatic vessels, and stimulating mitochondria of meibomian glands, thereby promoting cell activity and consecutive improvement of the meibomian gland microstructure. Its effects last 45 days to 27 months, depending on severity. However, side effects after IPL treatment were reported, including cheek swelling, conjunctival cysts, skin blistering, and hair loss at the brow and forehead [43]. Compared with these two device-based therapies, QMR has fewer limitations of use and fewer side effects, and the impact at the transcriptional level may persist longer after QMR treatment. The longest study confirmed that transcutaneous electrical stimulation (TES), which shares similar principles with QMR, is effective in improving dry eye disease over a 12-month period [5].

The phase-transition temperature of MGD (35 °C) is higher than that of healthy meibomian glands (32–33 °C) [44]. Thus, heat-based MGD treatment requires at least 40–45 °C for eyelid compression to melt pathologically altered meibum [2]. In our study, the lid temperature significantly increased only by 1.50–1.77 °C after QMR treatment. Therefore, the QMR-induced increase in lid temperature may not be important for meibum quality improvement.

Regarding the QMR safety profile, BCVA, UCVA, and IOP did not change throughout the study. Despite an increased eyelid skin temperature after treatment, adverse events like upper eyelid redness were mild and resolved without treatment within 3 days. Moreover, ocular examinations did not reveal any abnormalities throughout the study.

Our study had some limitations. First, our evaluations may not have been frequent enough. This could have affected our findings regarding certain parameters, including TMH, NITBUT, TFLLT, tear osmolarity, Schirmer’s test, lid margin irregularity, and meibum expressibility. Second, a longer follow-up time might be needed to show the changes of some parameters. Third, because the microbiome and *Demodex* mites were found to be associated with MGD [2], further studies on the effects of bacteria and *Demodex* load are needed. Finally, seven participants were lost to follow-up in this study (two and five participants of the QMR and sham-QMR groups, respectively); more participants might be needed to identify adverse QMR effects. However, the number of participants exceeded the calculated minimum sample size.

## Conclusion

In conclusion, the use of a QMR-based device is a promising and less invasive alternative to MGD treatment. It improved meibum quality and alleviated inflammation of the ocular surface, as demonstrated by clinical improvements of the cornea and conjunctival staining and decreased IL-6 levels in tears.

## Summary

### What was known before

- Quantum molecular resonance could be used as an alternative treatment for meibomian gland dysfunction; however, there is no previous randomized controlled trial of quantum molecular resonance and meibomian gland dysfunction.

### What this study adds

- This study is the first randomized controlled trial study which showed that quantum molecular resonance (QMR) could help meibomian gland dysfunction (MGD). QMR showed a significant reduction in IL-6 cytokine in the tear and facilitated meibomian gland regeneration. This study found that meibomian gland plugging grade and telangiectatic vessels of the lid margin in MGD patients were significantly decreased after QMR treatment; however, there was no improvement in tear film lipid layer thickness, tear osmolarity, bulbar conjunctival hyperemia, lid margin thickening, and irregularity grade after QMR treatment.

## Supplementary information

Supplementary Figure 1

Supplementary Figure 2

Supplementary Figure 3

Supplementary Data

## Data Availability

Datasets analyzed in this study are available on reasonable request.
