# Supplementary figures and images for: Randomized, double-masked, sham-controlled trial of efficacy and safety of quantum molecular resonance for treating meibomian gland dysfunction
Source: Eye (Lond). 2025 Jun 27;39(12):2451–9. doi: 10.1038/s41433-025-03890-3 (PMC12325607; doi:10.1038/s41433-025-03890-3)

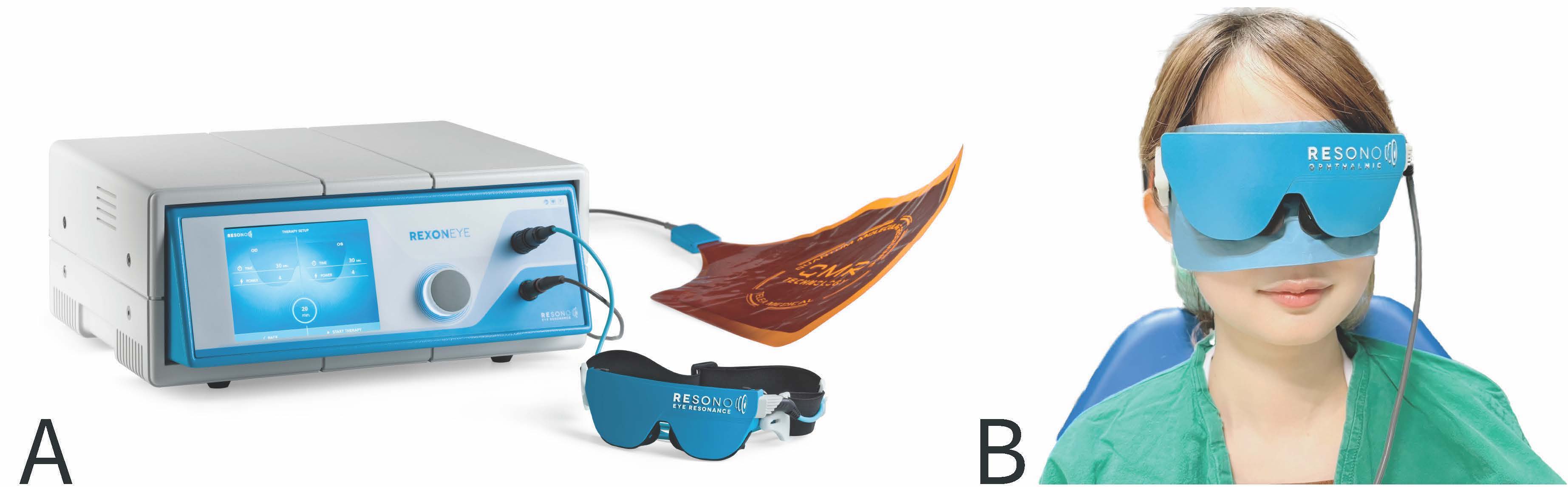

Supplement: Supplementary file 1 — Supplementary Figure 1 [file 41433_2025_3890_MOESM1_ESM.jpg]

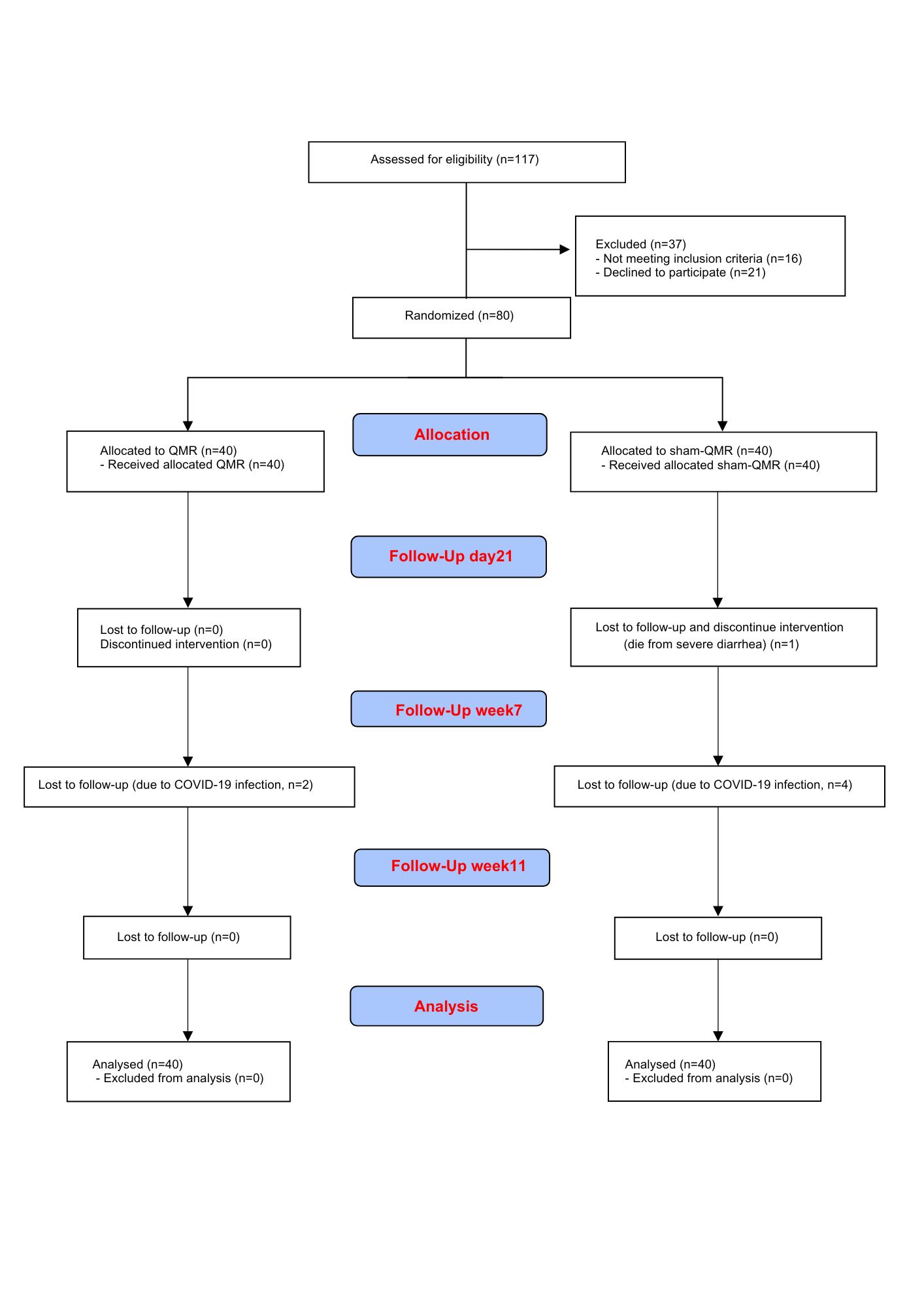

Supplement: Supplementary file 2 — Supplementary Figure 2 [file 41433_2025_3890_MOESM2_ESM.jpg]

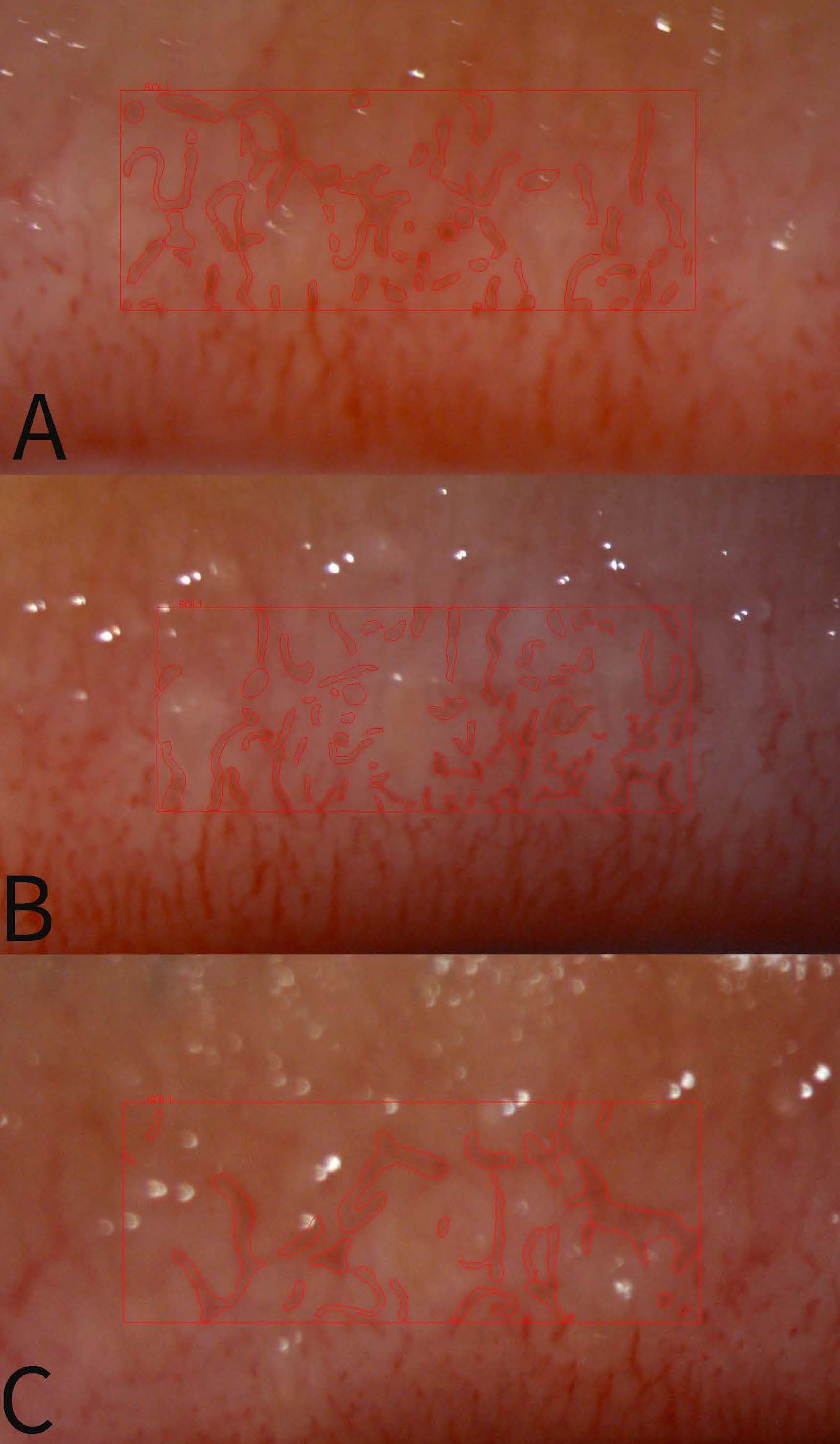

Supplement: Supplementary file 3 — Supplementary Figure 3 [file 41433_2025_3890_MOESM3_ESM.jpg]
