# Supplementary material for: Randomized, double-masked, sham-controlled trial of efficacy and safety of quantum molecular resonance for treating meibomian gland dysfunction
Source: Eye (Lond). 2025 Jun 27;39(12):2451–9. doi: 10.1038/s41433-025-03890-3 (PMC12325607; doi:10.1038/s41433-025-03890-3)
Supplement: Supplementary file 4 — Supplementary Data [file 41433_2025_3890_MOESM4_ESM.docx]

**Supplementary data**

Exclusion criteria included

1) to limit confounders, 1a) contact lens use within the past 1 month and throughout the study, 1b) ocular or eyelid surgery within the past 6 months, 1c) neuro-paralysis in the treatment area within the past 6 months, 1d) refractive surgery within the past 6 months, 1e) intense pulse light (IPL) therapy, thermal pulsation treatment, or any equivalent treatment within the past 12 months, 1f) any anti-glaucomatous eye drop use within the past 3 months and throughout the study, and 1g) any active ocular inflammation and 2) for safety reasons, 2a) pre-cancerous lesions or skin cancer in the planned treatment area, 2b) uncontrolled infections or immunosuppressive diseases, 2c) pregnancy and lactation, 2d) radiation therapy to the head or neck within the past year or planned radiation therapy throughout the study period, 2e) legally blind in one eye, and 2f) any other condition deemed inappropriate by the treating physician.

Clinical assessment

The sequence was ordered from noninvasive to most invasive procedures (Ocular Surface Disease Index score [OSDI], uncorrected visual acuity, best-corrected visual acuity, tear meniscus height [TMH], noninvasive tear break-up time [NITBUT], bulbar conjunctival hyperemia, tear film lipid layer thickness [TFLLT], tear osmolarity, corneal and conjunctival fluorescein staining score, Schirmer’s test, lid telangiectasia, lid margin thickening and irregularity grade, meibomian gland plugging grade, superior and inferior lid meiboscales, meibum quality score and expressibility grade, and intraocular pressure).^1, 2^ The temperature of the upper eyelid skin was recorded before and immediately after treatment on days 0, 7, 14, and 21 using an infrared thermometer. Participants were instructed to report any uncomfortable events during and after treatment to the research assistant. The OSDI score based on the 12-item dry eye questionnaire was recorded by well-trained research assistants. We recorded NITBUT from an average of three measurements, TMH, bulbar conjunctival hyperemia, and superior and inferior lid meiboscales were measured using a Keratograph 5M (OCULUS, Wetzlar, Hessen, Germany). Bulbar conjunctival hyperemia was graded using the JENVIS grading scale (0, no finding; 1, single injection; 2, mild diffuse injection; 3, severe local injection; and 4, severe diffuse injection). The Keratograph also visualized the morphology of the meibomian glands of both the upper and lower lids *in vivo* using a noncontact, Placido ring-based corneal topographer. This allowed us to grade the degree of meibomian gland dropout (meiboscale) as follows: grade 0 (no gland loss), grade 1 (≤25%), grade 2 (26–50%), grade 3 (51–75%), and grade 4 (≥75% loss).^3^ We obtained TFLLT using a tear film interferometer (LipiView II ^®^Ocular Surface Interferometer; TearScience, Morrisville, NC, USA), which analyzed the image of the surface contour of the tear film. Tear osmolarity was measured using the TearLab^TM^ Osmolarity System (Escondido, CA, USA). This test required tear fluid to be collected directly from the eyelid margin using the Test Card held by the TearLab Pen. Corneal and conjunctival fluorescein stainings were graded using the Oxford staining score, obtained under cobalt blue light after fluorescein staining with a FLUORO strip (Contacare Ophthalmics and Diagnostics, Vadodara, Gujarat, India). Staining severity was graded 0–5 in the cornea and two conjunctival areas,^1^ resulting in an overall staining score of 0–15. The Schirmer I test was performed without anesthetic eye drops by placing Schirmer strips MARK BLU Tear Test^®^ strips (Optitech Eyecare, Allahabad, Uttar Pradesh, India) at the inferior fornix. After 5 min, the wetness of the filter paper was measured in mm from the initial fold. All Schirmer strips were collected in 2 mL centrifuge tubes at 4°C and then kept at -20°C until evaluation for cytokines .^4^ The cytokines (IL1-Ra and IL-6) were measured using the Bio-Plex® 200 system (Bio-Rad, Hercules, CA, USA). Photographs of upper lid telangiectasia were recorded using IMAGEnet R4^TM^ (Topcon Corporation, Tokyo, Japan). All images were analyzed using the cellSens Dimension software (Olympus, Hamburg, Germany). The area of interest was the central 1/5 of the total upper lid length, and telangiectatic vessels were recorded in pixels which refered to the size of individual pixels in an image which is the unit of size from cellSens. Both upper and lower eyelids were evaluated and graded. Based on Arita et al.,^5^ lid margin thickening was classified into three grades (0, no lid margin thickening; 1, lid margin thickening with or without localized rounding; and 2, lid margin thickening with diffuse rounding). Lid irregularity was graded as follows: 0, no lid margin irregularity; 1, <3 lid margin irregularities with shallow notching; and 2, ≥3 lid margin irregularities or deep notching. Meibomian gland plugging was graded as 0, no plugged gland orifices; 1, <3 plugged gland orifices; 2, ≥3 plugged gland orifices distributed over less than half of the entire lid length; and 3, ≥3 plugged gland orifices distributed over at least half of the entire lid length. Meibum quality was measured using meibomian gland expression forceps. The meibum quality score was assessed at the central third of eight meibomian glands of the lower lid, with each gland rated on a scale comprising 0, clear fluid; 1, cloudy liquid; 2, cloudy; and 3, inspissated/toothpaste-like.^6^ Meibum expressibility was assessed at the central five glands of the lower lid and graded 0–3 according to the number of expressible glands (0, all glands; 1, 3–4 glands; 2, 1–2 glands; and 3, no glands).^6^

**Supplementary table 1.** Baseline characteristics and clinical parameters of both quantum molecular resonance and sham-treated groups.

| **Baseline characteristic** | **QMR (N=40)** | **Sham-QMR (N=40)** |
| --- | --- | --- |
| **Age: mean (SD)** | 57.97 (11.40) | 59.60 (12.05) |
| **Gender (N, %)** | | |
| Male | 12 (30%) | 13 (32.50%) |
| Female | 28 (70%) | 27 (67.50%) |
| **Side (N, %)** | | |
| Right eye | 19 (47.50%) | 26 (65%) |
| Left eye | 21 (52.50%) | 14 (35%) |
| **Group MGD (N, %)** | | |
| Group1 (MGD stage1+2) | 20 (50%) | 17 (42.50%) |
| Group2 (MGD stage3+4) | 20 (50%) | 23 (57.50%) |
| **Baseline parameters (mean, SD or median, Q1-Q3)** | | |
| OSDI score (0-100) | 24.67 (19.69) | 30.74 (20.57) |
| TMH (mm) | 0.16 (0.12, 0.20) | 0.16 (0.13, 0.20) |
| NITBUT (sec) | 7.87 (5.08, 12.79) | 7.49 (5.75, 11.34) |
| Bulbar conjunctival hyperemia  (JENVIS grading scale, 0-4) | 1.32 (0.42) | 1.31 (0.45) |
| Tear film lipid layer thickness (nm) | 60.12 (28.21) | 49.72 (31.27) |
| Tear osmolarity (mOsm/L) | 297.86 (9.70) | 297.32 (13.36) |
| Corneal and conjunctival fluorescein staining score (0-15) | 2.28 (2.00) | 1.67 (1.47) |
| Schirmer test (mm) | 12.73 (9.90) | 18.27 (12.60) |
| Lid telangiectasia (pixel) | 33140.97 (13560.61) | 25706.50 (15313.73) |
| Lid margin thickening grade (0-2) | 1.23 (0.54) | 1.23 (0.66) |
| Lid margin irregularity grade (0-2) | 1.03 (0.78) | 1.20 (0.76) |
| MG plugging grade (0-3) | 2.44 (0.94) | 2.33 (0.92) |
| Superior lid meiboscale (0-4) | 1.60 (0.87) | 1.97 (1.01) |
| Inferior lid meiboscale (0-4) | 1.05 (0.78) | 1.40 (0.93) |
| Meibum quality score (0-24) | 16.10 (4.25) | 16.40 (3.71) |
| Meibum expressibility grade (0-4) | 1 (0, 1.5) | 1 (0, 2) |
| Best-corrected visual acuity (logMAR) | 0.07 (0.11) | 0.09 (0.12) |
| Uncorrected visual acuity (logMAR) | 0.25 (0.27) | 0.30 (0.34) |
| Intraocular pressure (mmHg) | 14.98 (2.82) | 15.20 (2.79) |
| **Tear cytokine level (GM, %CV)** | | |
| IL-6 (pg/ml) | 29.63 (375.49) | 18.15 (316.43) |
| IL-Ra (pg/ml) | 296,155 (366.91) | 208,050.8 (284.41) |

%CV, coefficient of variation; GM, geometric mean; MGD, meibomian gland dysfunction; NITBUT, noninvasive tear break-up time; OSDI, Ocular Surface Disease Index; TMH, tear meniscus height.

| Variables  (mean; 95% confidence interval) | **QMR-Sham group** | **QMR group** |
| --- | --- | --- |
| **BCVA** | | |
| Day 0 | 0.09 (0.07, 1.11) | 0.07 (0.05,0.09) |
| Day 7 | 0.06 (0.04,0.08) | 0.04 (0.02, 0.06) |
| Day 14 | 0.07 (0.05,0.10) | 0.05 (0.03, 0.07) |
| Day 21 | 0.07 (0.04,0.09) | 0.04 (0.02,0.07) |
| Week 7 | 0.08 (0.06,0.10) | 0.06 (0.03,0.08) |
| Week 11 | 0.08 (0.06,0.10) | 0.06 (0.03,0.08) |
| **UCVA** | | |
| Day 0 | 0.28 (0.24,0.32) | 0.27 (0.23,0.31) |
| Day 7 | 0.26 (0.22,0.30) | 0.26 (0.21,0.30) |
| Day 14 | 0.24 (0.20,0.28) | 0.24 (0.19,0.28) |
| Day 21 | 0.26 (0.21,0.30) | 0.25 (0.21,0.29) |
| Week 7 | 0.27 (0.23, 0.31) | 0.26 (0.22,0.31) |
| Week 11 | 0.25 (0.20,0.29) | 0.24 (0.20,0.28) |
| **IOP** | | |
| Day 0 | 15.07  (14.62, 15.53) | 15.20 (14.74,15.65) |
| Day 7 | 14.73 (14.28,15.19) | 14.86 (14.40,15.32) |
| Day 14 | 14.40 (13.94,14.86) | 14.52 (14.06,14.98) |
| Day 21 | 14.42 (13.96,14.88) | 14.54 (14.08,15.00) |
| Week 7 | 14.47 (13.99,14.94) | 14.59 (14.12,15.06) |
| Week 11 | 14.16 (13.70,14.62) | 14.28 (13.83,14.74) |

**Supplementary table 2.** Best-corrected visual acuity, uncorrected visual acuity, and intraocular pressure at every visit during the study

**Supplementary table 3.** Mean temperatures of the QMR and sham-QMR groups before and after treatment at baseline and on days 7, 14, and 21.

| **Observed Temperature**  **(Celcius)** | **Before or after treatment** | **QMR**  **(mean**$\boldsymbol{\pm}$ **SD)** | **Sham-QMR**  **(mean**$\boldsymbol{\pm}$ **SD)** |
| --- | --- | --- | --- |
| Day 0 | Before | 34.84$\pm$5.42 | 35.66$\pm$1.35 |
|  | After | 37.30$\pm$1.48 | 35.90$\pm$1.37 |
| Day 7 | Before | 35.61$\pm$0.91 | 35.52$\pm$1.01 |
|  | After | 37.21$\pm$0.87 | 35.62$\pm$1.11 |
| Day 14 | Before | 34.95$\pm$1.28 | 35.47$\pm$1.37 |
|  | After | 36.70$\pm$1.29 | 35.83$\pm$1.23 |
| Day 21 | Before | 35.71$\pm$0.92 | 35.63$\pm$0.94 |
|  | After | 37.11$\pm$0.67 | 35.79$\pm$0.96 |

SD, standard deviation.

# Supplementary figure legends

Supplementary Fig. 1**.** Rexon-Eye device. (A) Electrical generator of the Rexon-Eye device with goggle electrode and neutral plate electrode. (B) Goggle electrode on the periocular area of a participant.

Supplementary Fig. 2**.** Consort flow diagram of this study.

QMR, quantum molecular resonance.

Supplementary Fig. 3. Improvement in the area of the lid margin telangiectasia vessels of a participant in the quantum molecular resonance group. Area at (A) baseline (before quantum molecular resonance treatment), (B) 7-week follow-up, and (C) 11-week follow-up.

**Reference**

1. Wolffsohn JS, Arita R, Chalmers R, et al. TFOS DEWS II Diagnostic Methodology report. *Ocul Surf* 2017;15:539-574.

2. Nichols KK, Foulks GN, Bron AJ, et al. The international workshop on meibomian gland dysfunction: executive summary. *Invest Ophthalmol Vis Sci* 2011;52:1922-1929.

3. Pult H, Riede-Pult B. Comparison of subjective grading and objective assessment in meibography. *Cont Lens Anterior Eye* 2013;36:22-27.

4. VanDerMeid KR, Su SP, Krenzer KL, Ward KW, Zhang JZ. A method to extract cytokines and matrix metalloproteinases from Schirmer strips and analyze using Luminex. *Molecular vision* 2011;17:1056-1063.

5. Arita R, Minoura I, Morishige N, et al. Development of Definitive and Reliable Grading Scales for Meibomian Gland Dysfunction. *Am J Ophthalmol* 2016;169:125-137.

6. Geerling G, Tauber J, Baudouin C, et al. The international workshop on meibomian gland dysfunction: report of the subcommittee on management and treatment of meibomian gland dysfunction. *Invest Ophthalmol Vis Sci* 2011;52:2050-2064.
